# Carbosilane Dendrimers Loaded with siRNA Targeting Nrf2 as a Tool to Overcome Cisplatin Chemoresistance in Bladder Cancer Cells

**DOI:** 10.3390/antiox9100993

**Published:** 2020-10-14

**Authors:** Leanne Ambrosio, Monica Argenziano, Marie Angèle Cucci, Margherita Grattarola, Inge A.M. de Graaf, Chiara Dianzani, Giuseppina Barrera, Javier Sánchez Nieves, Rafael Gomez, Roberta Cavalli, Stefania Pizzimenti

**Affiliations:** 1 Department of Clinical and Biological Science, University of Turin, 10125 Turin, Italy; l.ambrosio@student.rug.nl (L.A.); marieangele.cucci@unito.it (M.A.C.); margherita.grattarola@unito.it (M.G.); giuseppina.barrera@unito.it (G.B.); 2 Graduate School of Science, Faculty of Science and Engineering, University of Groningen, 9700 AB Groningen, The Netherlands; i.a.m.de.graaf@rug.nl; 3 Department of Scienza e Tecnologia del Farmaco, University of Turin, 10125 Turin, Italy; monica.argenziano@unito.it (M.A.); chiara.dianzani@unito.it (C.D.); roberta.cavalli@unito.it (R.C.); 4 Department of Organic Chemistry and Inorganic Chemistry, Research Institute in Chemistry “Andrés M. Del Río” (IQAR), University Campus, University of Alcalá, Alcalá de Henares E-2885 Madrid, Spain; javier.sancheznieves@uah.es (J.S.N.); rafael.gomez@uah.es (R.G.); 5 Networking Research Center on Bioengineering, Biomaterials and Nanomedicine (CIBER-BBN), 28029 Madrid, Spain; 6 Ramón y Cajal Health Research Institute (IRYCIS), 28034 Madrid, Spain

**Keywords:** carbosilane dendrimers, small interfering RNA (siRNA), nuclear factor erythroid 2-related factor 2 (Nrf2), bladder cancer, cisplatin resistance, non-cancerous HK-2 cells

## Abstract

The transcription factor nuclear factor erythroid 2-related factor 2 (Nrf2) is considered as the master regulator of antioxidant and cytoprotective gene expressions. Moreover, it plays a pivotal role in cancer progression. Nrf2 mediates the adaptive response which contributes to the resistance to chemotherapeutic pro-oxidant drugs, such as cisplatin (CDDP), in various tumors, including bladder cancers. For this reason, Nrf2 could be a promising target to overcome chemoresistance. There are several known Nrf2 pharmacological inhibitors; however, most of them are not specific. The use of a specific small interfering RNA (siRNA) targeting the Nrf2 gene (siNrf2) loaded into nanovehicles is an attractive alternative, since it can increase specificity. This study aimed to evaluate the biological activity of siNrf2 loaded on guanidine-terminated carbosilane dendrimers (GCDs) in overcoming CDDP resistance in bladder cancer cells with a high level of Nrf2. Parameters such as viability, proliferation, apoptosis, migration, and oxidative stress level were taken into account. Results demonstrated that siNrf2-GCD treatment sensitized CDDP-resistant cells to CDDP treatment. Moreover, data obtained by treating the non-cancerous human kidney HK-2 cell line strongly suggest a good safety profile of the carbosilane dendrimers loaded with siNrf2. In conclusion, we suggest that siNrf2-GCD is a promising drug delivery system for gene therapy to be used in vivo; and it may represent an important tool in the therapy of CDDP-resistant cancer.

## 1. Introduction

The nuclear factor E2-related factor 2 (Nrf2) seems to play a pivotal role in drug resistance in several types of tumors, including bladder cancer. The transcription factor Nrf2 is considered the master regulator of antioxidant and cytoprotective gene expressions [1]. In physiological conditions, Nrf2 is found in the cytosol linked to its inhibitor, the Kelch-like erythroid cell-derived protein with Cap ‘n’ collar homology [ECH]-associated protein (Keap1), leading to inactivation via ubiquitination and proteasomal degradation. In response to an oxidative stimulus, Keap1 undergoes a conformational change and releases Nrf2. Nrf2 then translocates into the nucleus and binds to the antioxidant response elements (ARE) sequences present in the promoter of genes coding for antioxidant enzymes, such as heme oxygenase-1 (HO-1) and numerous genes related to glutathione (GSH) metabolism, such as γ-glutamate-cysteine ligase and glutathione-S-transferase A4 (GSTA4) [1]. Initially, due to its ability to detoxify carcinogens and protect cells from oxidative stress damage, a protective role was attributed to Nrf2 in the early stages of malignant transformation. However, more recent data showed an opposite role in the advanced stages of the cancer disease, since aberrant activation of Nrf2 is involved in tumor progression, not only by affecting chemoresistance and radioresistance of various malignant cancers, but also by modulating epithelial-mesenchymal transition (EMT), migration, invasion, and angiogenesis [1,2,3].

In various types of cancers, it has been shown that the adaptive response mediated by Nrf2 contributes to the resistance towards chemotherapeutic pro-oxidant drugs, such as cisplatin (CDDP). Recently, we provided evidence on its key role in sustaining CDDP resistance in bladder cancer [4,5,6], in agreement with other reports [7]. For these reasons, Nrf2 can be a promising target to overcome chemoresistance.

Unfortunately, although there are several known Nrf2 pharmacological inhibitors, most of them are not very specific [8,9]. Besides brusatol, a plant extract able to inhibit Nrf2 expression transiently [10], we have recently demonstrated that ailanthone, a quassinoid extracted from the Ailanthus altissima plant, is a potent inhibitor of Nrf2, able to overcome the chemoresistance in bladder cancer [5,6]. However, both brusatol and ailanthone could produce further nonspecific responses, likely due to the off-target effects [6,11]. The use of a specific small interfering RNA (siRNA) targeting the Nrf2 gene is an attractive alternative, since it could entail greater specificity compared to pharmacological molecules. However, siRNAs are unstable in blood and have very poor ability to cross the lipophilic cell membranes [12]. Therefore, the use of a nanostructure delivery system appears to be a good strategy to overcome these major impediments and to allow their possible clinical applications [13]. Nanovehicles can protect siRNA from degradation during systemic circulation and transport siRNA to target cells avoiding nonspecific delivery [14]. Moreover, through the EPR (enhanced permeability and retention) effect, these particles can preferably accumulate in the tumor site, providing prolonged drug release [15,16].

Among the nanovehicles, cationic carbosilane dendrimers represent a promising nanotechnology vector for the therapeutic administration of siRNAs [17,18]. Dendrimers are highly branched monodisperse macromolecules, with a well-defined globular shape, that are synthesized by controlled step-by-step procedures [19]. Different types of cationic groups can be introduced on the surface of carbosilane dendrimers, for example, ammonium or guanidine, so that they can form electrostatic interaction with the negatively charged nucleic acids, such as siRNA [20].

This study aimed to evaluate the biological activity of siRNA targeting Nrf2, loaded in a guanidine-terminated carbosilane dendrimer (GCD), in reducing CDDP resistance in bladder cancer cells with a high level of Nrf2, by analyzing parameters such as viability, proliferation, apoptosis, migration, and oxidative stress level.

## 2. Material and Methods

### 2.1. Preparation and Physico-Chemical Characterization of Guanidine-Terminated Carbosilane Dendrimers Loaded with siRNA Targeting Nrf2 (siNrf2-GCD) or 6-Coumarin

**Figure 1:**
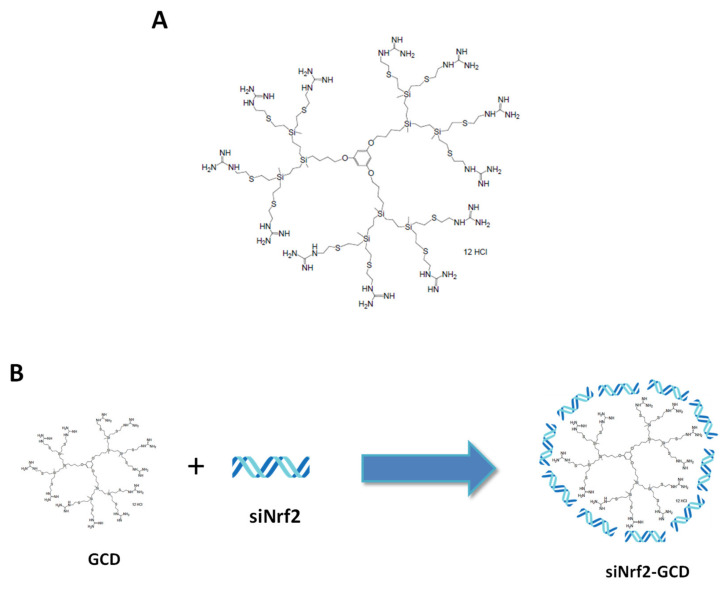
(**A**): Chemical structure of the guanidine-terminated carbosilane dendrimer (GCD). (**B**): Scheme of small interfering RNA targeting nuclear factor E2-related factor 2 (siNrf2)-loaded GCD (siNrf2-GCD) preparation. siNrf2 were added dropwise and under magnetic stirring to a GCD dendrimer suspension and then Pluronic F68^®^ 0.15% v/v was added.

For this study, we have used the guanidine-terminated carbosilane dendrimers, BDLS002, referred to as GCD, with twelve guanidinium groups located at the surface of the structure (Figure 1A), prepared in-house by Prof. Gomez (University of Alcala, Spain) as previously described [20].

To obtain blank-GCD, we resuspended its hygroscopic form in a Hepes-buffered saline solution (150 mM NaCl, 25 mM Hepes, pH 6), to a final concentration of 1 mg/mL. Pluronic F68^®^ (Merck Life Science S.r.l., Milan, Italy) (0.15% v/v) was added to the blank-GCD, to shield dendrimer charge.

Anti-Nrf2, siRNA-loaded dendrimers (siNrf2-GCD) were prepared by adding the anti-Nrf2 siRNA (SI03246950, Qiagen S.r.l., Milan, Italy) dropwise and under magnetic stirring to the previously obtained dendrimer suspension. Then, the samples were incubated under magnetic stirring in an ice bath for 30 min, to promote the electrostatic interaction between siRNA and dendrimer, as schematized in Figure 1B. After 30 min of incubation, Pluronic F68^®^ (0.15% v/v) was added to the siNrf2-GCD formulation. Fluorescent-labeled GCD were obtained incubating the blank-GCD nanosuspension with 6-coumarin (ratio 1:10.000 w:w 6-coumarin:GCD).

Subsequently, dendrimer formulations were physico-chemically characterized by measuring the average diameter, polydispersity index, and zeta potential, using dynamic light scattering as previously described [21]. Each reported value is the average of ten measurements of at least three different formulation batches.

Moreover, gel retardation assay using electrophoresis on agarose gel was performed to confirm the complexation between the anti-Nrf2 siRNA and the dendrimers. The DNA ladder (O’Range Rules 10bp DNA ladder, Thermo Fisher Scientific- Fisher Scientific Italia, Rodano, Milan, Italy) and the samples stained with an ethidium bromide solution (0.5 μg/mL) were loaded onto the agarose gel (4% w/v). The electrophoresis runs in TAE buffer (40 mM Tris base, 20 mm acetic acid, and 1 mm EDTA; pH 8.0) at 120 V for 40 min. The banding pattern was visualized using an ultraviolet transilluminator and photographed with a Polaroid camera.

### 2.2. Cell Lines

For this study, three human bladder cancer cell lines were used: T24 (purchased from ATCC, Manassas, VA, USA), with intrinsic resistance to CDDP, 253J B-V CDDP-sensitive cells, and the 253J B-V CDDP-resistant subclone (253J B-V C-r). Both 253J B-V and 253J B-V C-r cell lines were kindly provided by Dr. Roberto Pili from Roswell Parc Cancer Institute, Buffalo, United States of America. 253J B-V C-r cells were generated as already described [4]. In addition, as a model of non-cancerous cells, we have used the human kidney-2 (HK-2) cell line. All cell lines were cultured in RPMI 1640, except HK-2, which needs low glucose DMEM; all of them were supplemented with 10% fetal bovine serum (FBS) and antibiotics (100 units/ml of penicillin and 100 μg/mL streptomycin) in a 5% CO_2_, 37 °C incubator.

### 2.3. MTT

T24 (1,500 cells/wells), 253J B-V (2,500 cells/wells), 253J B-V C-r (2,500 cells/wells), and HK-2 (2000 cells/wells) were seeded in a 96-well plate with 200 μL of serum-supplemented medium. After cell treatments, the viability was assessed using MTT (3-(4,5-dimethyl thiazol-2-yL)-2,5-diphenyltetrazolium bromide) (Merck Life Science S.r.l.) assay, as previously reported [5].

### 2.4. Colony Forming Assay

Cells were seeded into a 6-well plate (1,000 cells/well) and left overnight to adhere to the surface. After 24 h, cells were treated, and cultured for 9 to 11 days. They were then fixed and stained with a solution of crystal violet (Sigma-Aldrich) and methanol (9:1 ratio). The colonies were photographed; afterwards, they were quantified by dissolving the violet staining in 10% acetic acid. Plates were placed for at least 15 min on a shaker to destain all the cells. Samples were diluted with purified water (1:4) and measured with a 96-well-plate ELISA reader (iMark Microplate Reader, Bio-Rad Laboratories S.r.l., Segrate, Milan, Italy) at 595 nm.

### 2.5. Apoptosis

T24 and 253J B-V C-r cells were seeded in 6-well plates (250,000 cells/well). After treatments, adherent and non-adherent cells were harvested, washed in 1 × PBS, and subsequently stained with annexin V, conjugated to the fluorescein isothiocyanate (FITC) dye, and propidium iodide (PI), according to the manufacturer protocol (FITC annexin V Apoptosis Detection Kit, Cat. No. 556547, BD Biosciences, Milan, Italy). Cells were analyzed using a FACScan cytometer Accuri C6 (Becton Dickinson Italia, Milan, Italy).

### 2.6. Western Blot

Western blot analysis was performed as already reported [4]. The following antibodies were used: β-actin (#4970S, Cell Signaling Technology, distributed by EuroClone, Pero, Milan, Italy), Nrf2 (sc-722, Santa Cruz Biotechnology, Heidelberg, Germany), GSTA4 (SAB1401164, Merck Life Science S.r.l.), and GAPDH (14C10, 2118, Merck Life Science S.r.l.). The detection of the bands was carried out after reaction with chemiluminescence reagents (Amersham ECL Prime Western Blotting Detection Reagent, GE Healthcare), using the instrument ChemiDocTM XRS + (Bio-Rad Laboratories S.r.l.), or through film (sc-201697 Santa Cruz Biotechnology) autoradiography. The signal intensities relative to the protein products were quantified using densitometric scanning with the ImageJ software [22].

### 2.7. siNrf2 Transfection with a Traditional Protocol

The effect of siNrf2-GCD was compared with that obtained with the naked siNrf2 (SI03246950, Qiagen S.r.l.) transfected with HiPerFect® Transfection Reagent (301705, Qiagen S.r.l.) with a traditional protocol, suggested by the manufacturer. Briefly, transfection with HiPerFect® was carried out in the culture medium containing serum, but not antibiotics. To allow complexation between the siNrf2 and HiPerFect® reagent, both were diluted in culture medium without serum and antibiotics and incubated for 10 min at room temperature. Complexes were added dropwise onto the cells, according to the manufacturer’s instruction. T24 and 253J B-V C-r CDDP-resistant cells and non-cancerous HK-2 cells were treated with the same amount of siRNA as used in the siNrf2-GCD treatment.

### 2.8. Wound-Healing Assay

The wound-healing assay was performed as previously described [23]. Briefly, T24 (125,000 cells/well) and 253J B-V C-r cells (225,000 cells/well) were plated onto 6-well plates and grown to confluence. The cell monolayers were wounded by scratching with a micropipette tip across the center of the well. After scratching, the wells were gently washed to remove the detached cells. Then, cells were replenished with fresh medium containing a low amount of serum (2% FBS) to minimize cell proliferation so that it did not interfere with the measurement of migration. After that, cells were treated with several compounds. At least five fields of each wound were photographed immediately after the scratch (T0) and after 24 h. The reduction in the width of the wound after 24 h, compared to T0, which was set at 100%, was then calculated using the ImageJ software [22].

### 2.9. Detection of the Intracellular Oxidative Stress Level

T24 and 253J B-V C-r CDDP-resistant cells were seeded (250,000 cells/well) in a 6 wells plate in 2 ml of serum-supplemented RPMI 1640 medium. After 24 h, cells were treated. The intracellular oxidative stress level was analyzed 24 h later. Cells were incubated for 30 min with 1 µM 2′-7′-dichlorodihydrofluorescein diacetate (DCF-DA) (Invitrogen, Carlsbad, CA, USA). The amount of the fluorescent product (2,7-dichlorodihydrofluorescein, DCF) was measured using a FACScan cytometer Accuri C6 (Becton Dickinson Italia) [6].

### 2.10. Statistical Analysis

With GraphPad InStat software (San Diego, CA, USA), we performed one-way ANOVA analysis, followed by the Bonferroni multiple comparison post-test, to evaluate differences between experimental groups. Values of *p* ≤ 0.05 were considered statistically significant.

## 3. Results

### 3.1. Preparation and Physicochemical Characterization of Guanidine-Terminated Carbosilane Dendrimer Formulations

The GCD formulations were synthesized according to a previously established protocol (20). The preparation method of GCD nanoformulations has been optimized in relation to their toxicity on T24 and 253J B-V C-r cell lines (data not shown), two CDDP-resistant bladder cancer cells with a high level of Nrf2, previously characterized in our laboratory [4]. Indeed, the addition of Pluronic F68^®^ to GCD significantly lowered cell toxicity (data not shown). Average diameters, polydispersity indices, and zeta potentials of unloaded blank-GCD, siNrf2-GCD, and 6-coumarin-GCD are reported in Table 1. Data about concentrations and pH are also reported.

The unloaded blank-GCD and 6-coumarin-GCD showed an average diameter of about 300 nm. After incubation with siRNA, a significant decrease in size of about 60% was observed.

The polydispersity index, a parameter of the nanoparticle size distribution, was also determined. All values range between 0.05 and 0.1, indicating a monodisperse distribution [24].

A decrease in the zeta potential was observed in siNrf2-GCD (2.72 ± 0.28 mV) with respect to the blank-GCD values (12.10 ± 0.13 mV), while 6-coumarin-GCD showed similar values (10.34 ± 2.11 mV) to the unloaded nanoformulation.

**Figure 2:**
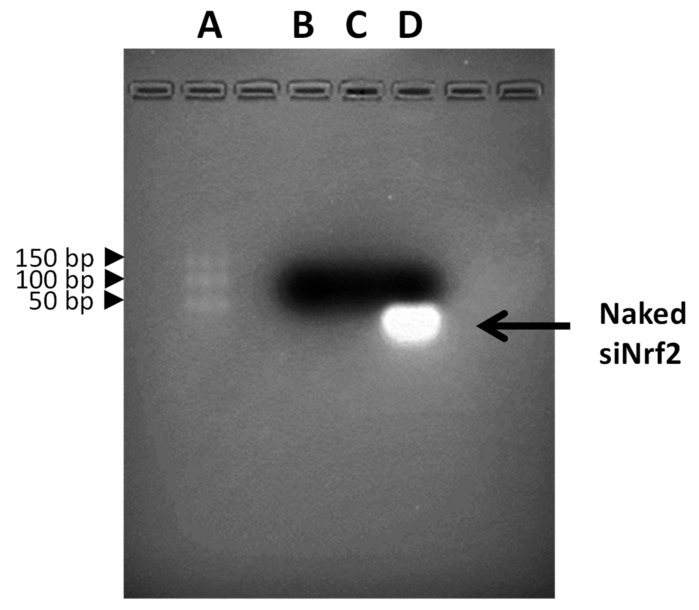
Electrophoresis of the GCD formulations. Lanes: (**A**) DNA ladder, (**B**) blank-GCD, (**C**) siNrf2-GCD, (**D**) naked siRNA.

The siRNA complexation with dendrimers was evaluated using gel retardation assay using electrophoresis on an agarose gel. In Figure 2 the results of the DNA ladder (lane A), blank-GCD (lane B), siNrf2-GCD (lane C), and naked siNrf2 (lane D) are reported. Naked siNrf2 ran towards the positive pole and it was visible on the gel; on the contrary, it was no longer visible in the agarose gel when loaded in GCD. The band disappearance confirmed the siRNA complexation with dendrimers.

### 3.2. Biological Effects of siNrf2-GCD in Bladder Cancer CDDP-Resistant Cells

#### 3.2.1. Cellular Penetration of GCD in CDDP-Resistant Bladder Cancer Cells

For this study, we chose T24 and 253J B-V C-r cells. Both cell lines are CDDP-resistant and express a high level of Nrf2 [4]. These features have been assessed (Figure S1), and the results are in agreement with our previous report [4].

**Figure 3:**
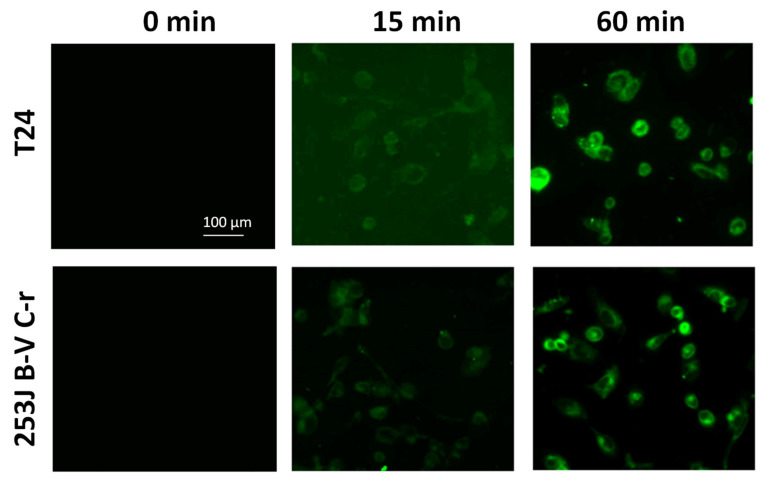
Fluorescent images of 6-coumarin-GCD uptake in T24 and 253J B-V C-r cells at the indicated time. Green fluorescence of 6-coumarin was examined using fluorescence microscopy (454 nm).

To assess the ability of carbosilane dendrimers to enter cells, we treated the CDDP-resistant cells with fluorescent-labeled 6-coumarin-GCDs (0.8 ng/mL 6-coumarin in 8 µg/mL GCD, final concentration) and observed using fluorescent microscopy. As shown in Figure 3, the fluorescent-labeled 6-coumarin-GCDs were internalized within 15 min into both cell types. After 60 min of incubation its accumulation significantly increased.

#### 3.2.2. siNrf2-GCD Down-Regulated the Expression of Nrf2 and its Target Gene GSTA4 in CDDP-Resistant Bladder Cancer Cells

**Figure 4:**
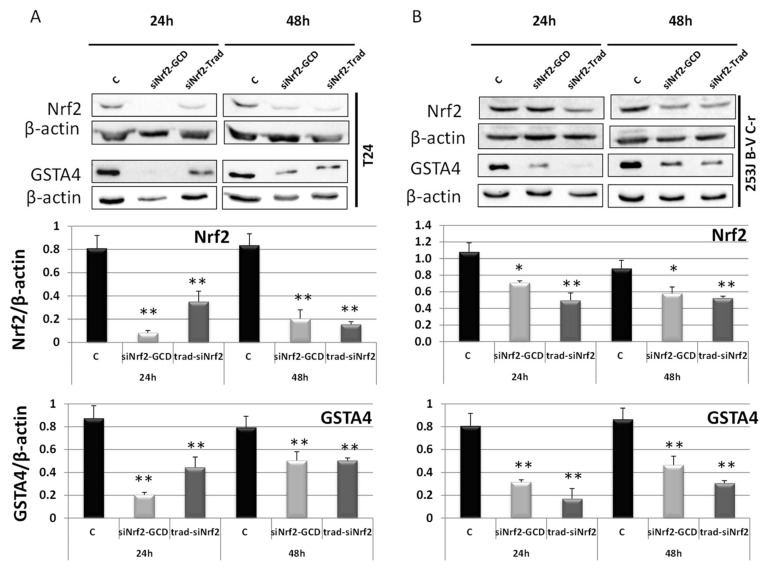
Western blot analysis of Nrf2 and GSTA4 in T24 (**A**) and 253J B-V C-r (**B**) untreated (control, C) treated with siNrf2-GCD (0.08 µM siNrf2 in 8 µg/mL GCD) or transfected with the same amount of siNrf2 (0.08 µM) with a traditional protocol (siNrf2-Trad). Cells were collected after 24 h and 48 h from the treatments. Below, relative densitometric scanning of Nrf2 and GSTA4 expression normalized using the β-actin signal. Data are the mean ± SD from three independent experiments. ** *p* ≤ 0.01 and * *p* ≤ 0.05 vs. control.

The ability of siNrf2-GCD to down-regulate the expression of the target gene Nrf2 was assayed using western blot analysis (Figure 4), at 24 h and 48 h after treatment in CDDP-resistant T24 and 253J B-V C-r cells. Both cell lines were treated with siNrf2-GCD alone (0.08 µM siNrf2 in 8 µg/mL GCD) or with 8 µg/mL naked siNrf2 transfected with a traditional protocol method. Results showed that Nrf2 expression was inhibited until 48 h in both cell lines. The reduction of Nrf2 expression also elicited an inhibition of its activity, which was demonstrated by the contemporary reduction of GSTA4, one of the gene targets of Nrf2. siNrf2 transfection with the traditional protocol method, used as a positive control, showed similar inhibitions.

#### 3.2.3. siNrf2-GCD Treatment Sensitized CDDP-Resistant Cells to CDDP Treatment: Effects on Viability, Proliferation, and Apoptosis

To evaluate the effects of siNrf2 silencing caused by siNrf2-GCD treatment in affecting CDDP resistance, viability and proliferation were assayed in T24 and 253J B-V C-r cells.

**Figure 5:**
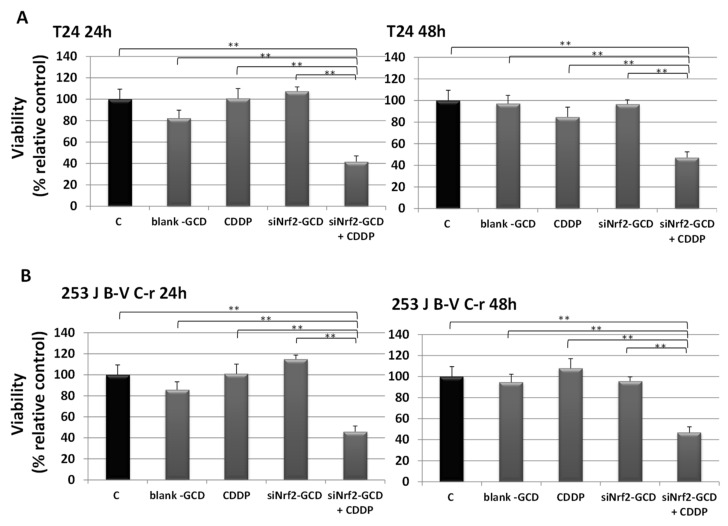
Viability was determined using MTT assay in T24 (**A**) and 253J B-V C-r (**B)** cells untreated (C, control) or treated with 8 µg/mL blank-GCD, 2.5 µg/mL CDDP, siNrf2-GCD (final concentrations 8 µg/mL GCD, 0.08 µM siNrf2), and siNrf2-GCD in combination with CDDP. Viability was measured at 24 h and 48 h. Results are expressed as percent of the relative control values and are the mean ± standard deviation of three separate experiments performed in triplicate. ** *p* < 0.01.

For viability, CDDP-resistant cells were exposed to 8 µg/mL blank-GCD, 2.5 µg/mL CDDP, siNrf2-GCD alone (0.08 µM siNrf2 in 8 µg/mL GCD), and siNrf2-GCD in combination with CDDP. After 24 h and 48 h, the MTT test was performed, and the results are shown in Figure 5. We observed a significant down-regulation on viability in cells treated with siNrf2-GCD in combination with CDDP. In contrast, treatments with blank-GCD, siNrf2-GCD, or CDDP alone were ineffective. The inhibition after the combined treatment was significant not only in comparison to control values, but also to those obtained after blank-GCD, siNrf2-GCD, or CDDP single treatments.

**Figure 6:**
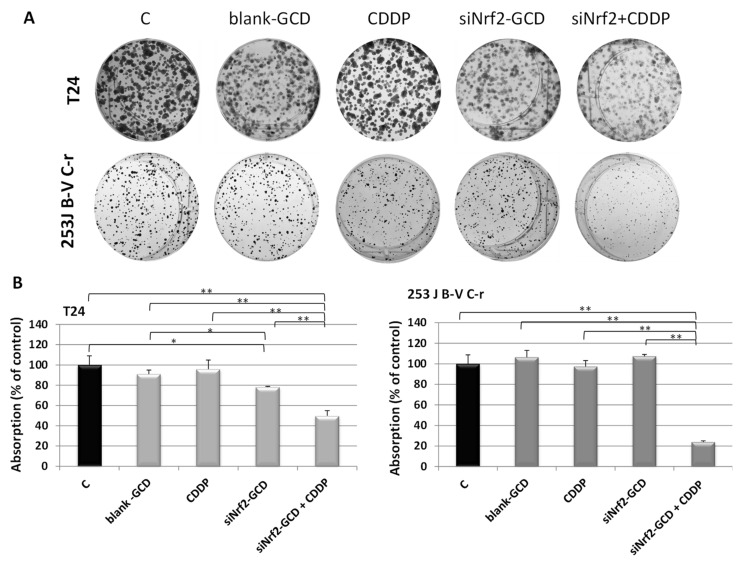
Colony-forming assay. (**A**): Representative images of cell colonies stained with crystal violet performed in T24 and 253J B-V C-r cells treated with 4 µg/mL blank-GCD, 0.1 µg/mL CDDP, siNrf2-GCD (0.04 µM siNrf2 in 4 µg/mL GCD), and siNrf2-GCD in combination with CDDP. (**B**): Quantitative analysis of colonies. Data are the mean ± standard deviation of three measurements. * *p* < 0.05; ** *p* < 0.01.

To evaluate proliferation, a colony-forming assay was performed. Both CDDP-resistant cells were exposed to 0.1 µg/mL CDDP, siNrf2-GCD (0.04 µM siNrf2 in 4 µg/mL GCD), and siNrf2-GCD in combination with CDDP. This experiment was performed after having confirmed the ability of this lower amount of siNrf2-GCD to down-regulate Nrf2 protein expression (Figure S2). As shown in Figure 6, siNrf2-GCD treatment sensitizes both cell lines to the CDDP treatments, leading to significant inhibition of cell proliferation. Blank-GCD or CDDP treatments alone were ineffective. Moreover, a slight but significant inhibition of proliferation was found in T24 cells, but not in 253J B-V C-r, treated with siNrf2-GCD alone. The inhibition of proliferation elicited by the combined treatment in both cell lines was significant in comparison to control values, and to those obtained by all the single treatments.

**Figure 7:**
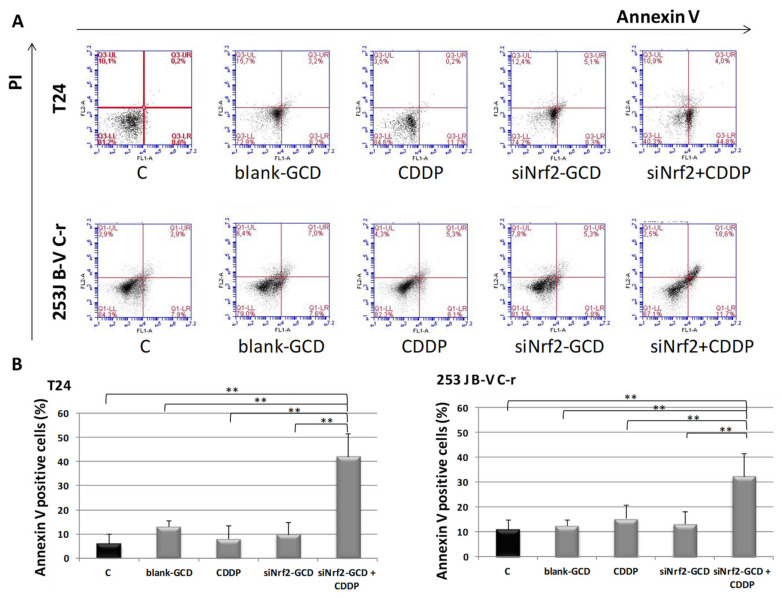
Apoptosis in T24, 253J B-V C-r cells untreated (C, control) or treated with 8 µg/ml blank-GCD, 2.5 µg/mL CDDP, siNrf2-GCD (0.08 µM siNrf2 in 8 µg/mL GCD), and siNrf2-GCD in combination with CDDP. Apoptosis was checked at 24 h via cytofluorimetric analysis of annexin V/PI stained cells. (**A**): Flow cytometry profiles of a representative experiment in annexin V/IP stained T24 and 253J B-V C-r cells at 24 h are shown. (**B**): Quantification of annexin V positive cells in T24 and 253J B-V C-r cells. Results are expressed as percent of the relative control values and are the mean ± standard deviation of three separate experiments. ** *p* < 0.01.

To measure the contribution of apoptosis to the observed viability inhibition, we performed an annexin V/IP assay in T24 and 253J B-V C-r cells under the same experimental conditions described above for the MTT test. After 24 h of treatment, we observed a significant increase of annexin V positive cells in both CDDP-resistant cell lines treated with siNrf2-GCD in combination with CDDP, while treatments with siNrf2-GCD or CDDP alone were ineffective (Figure 7).

#### 3.2.4. siNrf2-GCD Treatment Inhibited Migration In CDDP-Resistant Bladder Cancer Cells

In an attempt to investigate the effects of Nrf2 down-regulation caused by siNrf2-GCD on cell migration, a wound-healing assay was performed. CDDP-resistant cells were untreated (Control, C) or treated with non-toxic concentrations of blank-GCD (4 µg/mL) or siNrf2-GCD (0.04 µM siNrf2 in 4 µg/mL GCD). These doses were not able to affect cell viability, assessed using a MTT test. On the contrary, the combined treatment CDDP (0.1 µg/mL) and siNrf2-GCD (0.04 µM siNrf2 in 4 µg/mL GCD) significantly inhibited cell viability (data not shown), thus it was not suitable to evaluate cell migration.

**Figure 8:**
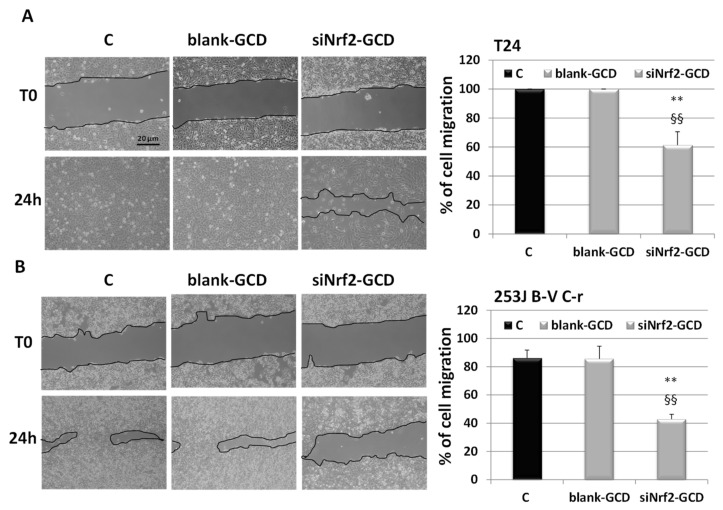
Cell migration. Wound healing assay at 0 (T0) and at 24 h in T24 (**A**), 253J C-r cells (**B**) untreated (C) or treated with blank-GCD (4 µg/mL), or siNrf2-GCD (0.04 µM siNrf2 in 4 µg/mL GCD). On the right, the quantification of wound healing is shown. The endpoint of the assay was measured by calculating the reduction in the width of the wound after 24 h and comparing it to T0, which is set at 100%. The data are the mean ± SD of three independent experiments. ** *p*-value ≤ 0.01 vs. C. §§ *p*-value ≤ 0.01 vs. blank-GCD.

After 24 h (Figure 8), we observed that the siNrf2-GCD-treated cells were able to migrate into the scratch slower than those treated with blank-GCD (around 60% and 40% with respect to the control in T24 and 253J C-r, respectively).

#### 3.2.5. siNrf2-GCD Treatment Enhanced Intracellular Oxidative Stress Caused by CDDP Treatments

**Figure 9:**
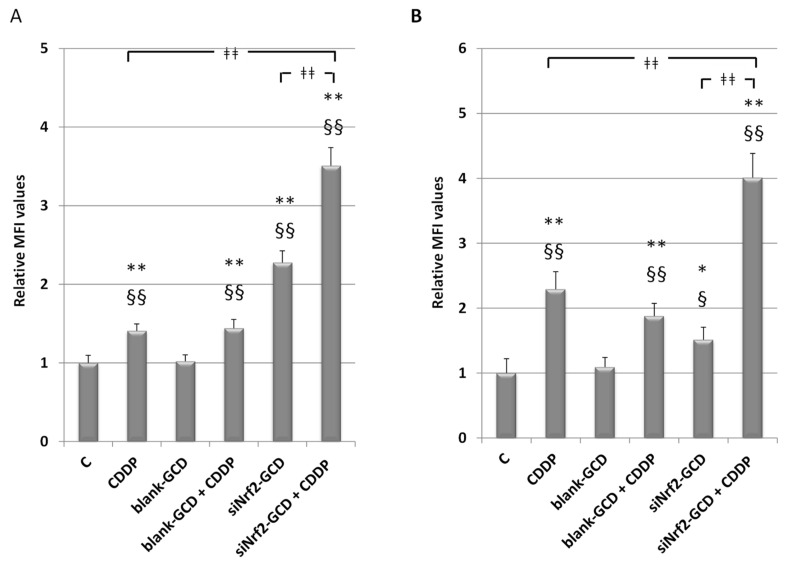
Intracellular oxidative stress level in T24 (**A**) and 253J B-V C-r (**B**) cells, 24 h after treatments with 2.5 µg/mL CDDP, 8 µg/ml blank-GCD, CDDP in combination with blank-GCD, siNrf2-GCD (0.08 µM siNrf2 in 8 µg/mL GCD), and siNrf2-GCD in combination with CDDP. The bar graphs show the relative values of the median fluorescence intensity (MFI), expressed as means ±  SD. * *p* < 0.05 vs. C; ** *p* < 0.01 vs. C; § *p* < 0.05 vs. blank-GCD; §§ *p* < 0.01 vs. blank-GCD; ǂǂǂǂ *p* < 0.01.

Since Nrf2 is one of the main regulators of the antioxidant response, we analyzed the effect of its down-regulation, caused by siNrf2-GCD treatment, on cellular redox status. Moreover, we aimed at evaluating if this treatment can further enhance the oxidative stress level induced by CDDP. Therefore, CDDP-resistant cells were untreated or treated with 2.5 µg/mL CDDP, 8 µg/mL blank-GCD, CDDP in combination with blank-GCD, siNrf2-GCD (0.08 µM siNrf2 in 8 µg/mL GCD), and siNrf2-GCD in combination with CDDP. As shown in Figure 9, at 24 h we observed an increase in the intracellular oxidative stress after CDDP treatments. Blank-CDG treatments were ineffective, while an increase was found in cells treated with both blank-GCD and CDDP, similar to that observed after CDDP treatment alone. The siNrf2-GCD treatments alone slightly enhanced oxidative stress, while the treatment with siNrf2-GCD in combination with CDDP elicited a great and significant increase of the intracellular oxidative stress level.

#### 3.2.6. Biological Effects in Non-Cancerous Human Kidney HK-2 Cells

To evaluate drug specificity or side effects, we treated HK-2 cells derived from normal human kidney with the same concentrations used in treating CDDP-resistant bladder cancer cells. HK-2 cells were obtained via a primary proximal tubular cell culture from normal adult human renal cortex and infected with a recombinant retrovirus containing HPV 16 E6/E7 genes. These cells show a phenotype as well as functional characteristics similar to the well-differentiated primary proximal tubular epithelium [25].

**Figure 10:**
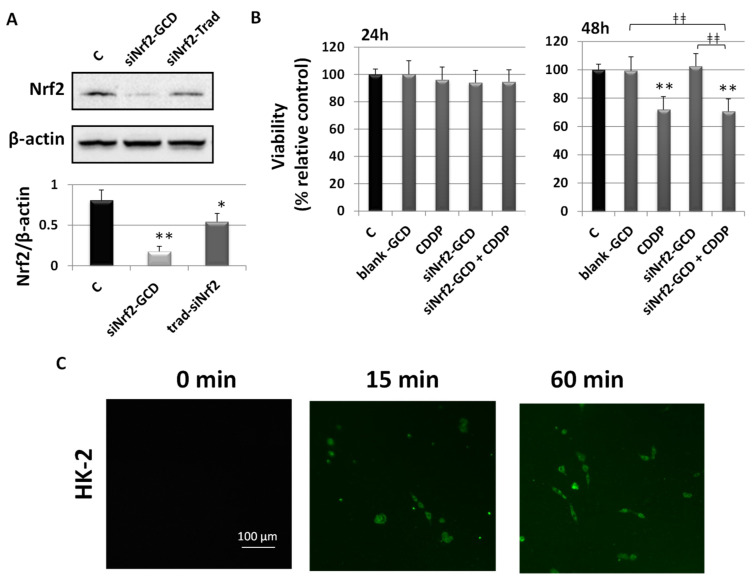
Biological effects in non-cancerous human kidney HK-2 cells. (**A**): Western blot analysis of Nrf2 in HK-2 cells untreated (control, C), treated with siNrf2-GCD (0.08 µM siNrf2 in 8 µg/mL GCD), or transfected with the same amount of siNrf2 (0.08 µM) with a traditional protocol (siNrf2-Trad). Cells were collected after 24 h and 48 h from the treatments. Below, relative densitometric scanning of Nrf2 expression normalized using the β-actin signal. Data are the mean ± SD from three independent experiments. ** *p* ≤ 0.01 and * *p* ≤ 0.05 vs. control. (**B**): Viability was determined using a MTT assay in HK-2 cells untreated (C, control) or treated with 8 µg/mL blank-GCD, 2.5 µg/mL CDDP, siNrf2-GCD (final concentrations 8 µg/mL GCD, 0.08 µM siNrf2), and siNrf2-GCD in combination with CDDP. Viability was measured at 24 h and 48 h. Results are expressed as percent of the relative control values and are the mean ± standard deviation of three separate experiments performed in triplicate. ** *p* < 0.01 vc. C; ǂǂǂǂ *p* < 0.01. (**C**): Fluorescent images of 6-coumarin-GCD uptake in HK-2 cells at the indicated time. Green fluorescence of 6-coumarin was examined by using fluorescence microscopy (454 nm).

The ability of siNrf2-GCD to down-regulate the expression of the target gene Nrf2 was assayed using western blot analysis (Figure 10, Panel A) at 24 h after treatment in HK-2 cells. This cell line was treated with siNrf2-GCD alone (0.08 µM siNrf2 in 8 µg/mL GCD) or with 8 µg/mL “naked” siNrf2 transfected with a traditional protocol method. Results showed that siNrf2-GCD was able to inhibit Nrf2. Transfection with the HiPerFect® reagent, used as a positive control, showed less inhibition, however significant compared to the control.

To evaluate the effect after siNrf2-GCD treatment on cell viability in HK-2 cells, a MTT assay was assessed after 24 h and 48 h (Figure 10, Panel B). HK-2 cells were exposed to 8 µg/mL blank-GCD, 2.5 µg/mL CDDP, siNrf2-GCD alone (0.08 µM siNrf2 in 8 µg/mL GCD), and siNrf2-GCD in combination with CDDP. Interestingly, at 24 h none of the treatments affected cell viability. At 48 h, we observed a significant inhibition after CDDP treatment alone (about 30%). A similar inhibition was observed after the treatment with siNrf2-GCD in combination with CDDP. 

To assess the ability of carbosilane dendrimers to enter cells, we treated HK-2 cells with fluorescent-labeled 6-coumarin-GCDs (0.8 ng/mL 6-coumarin in 8 µg/mL GCD, final concentration). Cells were observed using fluorescent microscopy. As shown in Figure 10, Panel C, the fluorescent-labeled 6-coumarin-GCDs were internalized within 15 min into HK-2 cells and remained after 60 min of incubation.

## 4. Discussion

A novel guanidine-terminated carbosilane dendrimer formulation carrying anti-Nrf2 siRNA was prepared. With the perspective of its in vivo use, this nanoformulation was not prepared with any toxic solvents, thus allowing a safe administration. Pluronic F68^®^, added during preparation to lower cell toxicity, is a suitable polymeric excipient admitted for oral and parental applications [26]. GCDs have a strong amphiphilic behavior, and this feature differentiates them from other kinds of dendrimers with a more hydrophilic skeleton; therefore, these nanocarriers are more prone to binding negatively charged oligonucleotides, such as siRNA, and transporting them to the interior of a range of cell types, assisted by the presence of lipophilic fragments [27,28]. Moreover, cationic carbosilane dendrimers have been successfully used for in vivo delivery of siRNA to the brain [29].

Physicochemical features showed that blank-GCD had an average diameter of 340 nm. However, after siNrf2 complexation, confirmed by electrophoresis on an agarose gel, a decrease in size is observed, reaching a diameter of around 130 nm. This shrinkage is likely due to a condensation of the polymer chain of the GCD via electrostatic interaction with siNrf2 [30]. This hypothesis is supported by the observed change of the zeta potential, which provides a measure of the dendrimer surface charge. Indeed, zeta potential in blank-GCD was around +10 mV, because of the terminal positive amino groups. After siNrf2 complexation, the values decreased, reaching around +2.7 mV; this indicates that an electrostatic interaction occurred between the negative phosphate groups of siNrf2 and the positively charged terminal groups of GCD along with the coating effect provided by the pluronic polymer.

All the PDI values of GCDs ranged between 0.05 and 0.1, indicating a homogenous population. These values are far below 0.3, which is considered the maximum acceptable in drug delivery applications using lipid-based carriers [31].

Efficient uptake of drug-loaded nanoparticles by cancer cells is needed to achieve effective drug delivery. The GCD nanoformulation rapidly entered the cells within 15 min, as demonstrated by results obtained with a fluorescent microscope, and it was able to inhibit both expression and activity of Nrf2. Of note, the use of a commercially available siRNA allowed us to minimize the risk of off-target effects.

The siNrf2-GCD concentration used during the experiments was able to down-regulate Nrf2 expression and activity, but it did not affect viability assayed using a MTT test in both CDPP-resistant cell lines. However, when combined with a non-toxic concentration of CDDP, we measured a significant inhibition of cell viability. These findings are in agreement with our previous results in T24 cells, where the siNrf2, transfected with a standard protocol, increased the sensitivity to CDDP treatment [4], as well as with the previous report on the CDDP-resistant RT112 bladder cancer subline [7].

We observed similar results for cell proliferation studies: siNrf2-GCD treatment sensitized both cell lines to the CDDP treatments, leading to significant inhibition of the colony formation. Moreover, T24 cells seem to be more sensitive than 253 J B-V C-r, since a slight but significant inhibition of proliferation was found after siNrf2-GCD treatment alone.

Apoptosis is likely to contribute to the decreased viability, since we observed a significant increase of annexin V positive cells in CDDP-resistant cells treated with a combined treatment of siNrf2-GCD and CDDP. It is very likely that cells died because of the great increase of oxidative stress, as demonstrated after siNrf2-GCD and CDDP combined treatment.

Nrf2 regulates not only the antioxidant capacity, but also the migration of cancer cells [1], which contributes to their metastatic properties. In agreement with these observations, siNrf2-GCD-treated CDDP-resistant cells migrated slower than blank-GCD-treated cells.

Nrf2 signaling is cross-talking with other important cell signaling involved in the control of cancer progression, such as Notch, Ras, and others [32,33]. Among them, recently we demonstrated cross-talk between Nrf2 and YAP in bladder cancer cells [4]. Therefore, we cannot exclude the contribution of YAP signaling or others in eliciting the observed biological effects.

Finally, data obtained by treating the non-cancerous human kidney HK-2 cell line suggests a good safety profile of the carbosilane dendrimers loaded with siNrf2. Indeed, although siNrf2-GCD was capable of inhibiting Nrf2 expression and entering quickly into cells (within 15 min), these events did not elicit toxic effects or increased susceptibility to cisplatin treatment. Results on carbosilane dendrimer uptake in HK-2 cells were similar to those obtained in CDDP-resistant bladder cancer cells. In both cases, fluorescent-labeled 6-coumarin-GCDs were internalized within 15 min and remained after 60 min of incubation. Although we did not perform qualitative assessments, at 15 min the fluorescence was more intense in HK-2 than in CDDP-resistant cells. This could be due to a different uptake rate, but also to the different cell sizes: HK-2 cells are much smaller than CDDP-resistant cells so that the cytoplasm could reach saturation faster.

In conclusion, the results presented here demonstrate that siNrf2-GCD treatment significantly increased the sensitivity to a pro-oxidant cytotoxic drug and reduced migration of CDDP-resistant bladder cancer cells. These data are in agreement with several reports [4,5,6,7] and further support the hypothesis that targeting Nrf2 is a means to overcome CDDP resistance in this tumor type. Although several natural, semisynthetic, or synthetic compounds have been identified as Nrf2 inhibitors, a lack of specificity is often observed [34]. Therefore, the use of specific siRNA can actually be a turning point. In 2018, the Food and Drug Administration and the European Medicines Agency approved the first-ever siRNA product loaded in a lipid-based nanoparticle for the treatment of a rare inherited condition, marking a significant milestone in the story of RNA interference (RNAi) [35]. On the other hand, dendrimers have already been approved for clinical application [36], and many clinical trials with dendrimers are now being conducted [37,38]. However, siRNA-loaded dendrimers to combat cancer disease are not yet approved for clinical purposes. In this context, cationic dendrimers, such as GCD, represent a promising drug delivery system for gene therapy to be used in vivo.

## Figures and Tables

**Table 1:** Physico-chemical characteristics of free and loaded GCD formulations.

Formulation	Average Diameter (nm) ^a^ ± SD	Polydispersity Index ^a^ ± SD	Zeta Potential (mV) ^a^ ± SD	pH
blank-GCD	346.8 ± 22.3	0.05 ± 0.005	12.10 ± 0.13	6.0
600 μg/mL GCD				
siNrf2-GCD	128.1 ± 10.1 **	0.09 ± 0.004 **	2.72 ± 0.28 **	6.0
6 µM siNrf2 in 600 μg/mL GCD				
6-coumarin-GCD	325.6 ± 20.34 **	0.110 ± 0.02 **	10.34 ± 2.11	6.0
0.06 μg/mL 6-coumarin				
in 600 μg/mL GCD				

^a^ Values represent mean ± SD (n = 3); ** *p* < 0.01 vs. blank-GCD.

## Supplementary Materials

The following are available online at https://www.mdpi.com/2076-3921/9/10/993/s1, Figure S1: CDDP-resistance and Nrf2 expression in bladder cancer cells, Figure S2: Nrf2 inhibition with a lower amount of siNrf2-GCD.

## References

[1] Rojo de la Vega M., Chapman E., Zhang D.D. (2018). NRF2 and the Hallmarks of Cancer. Cancer Cell.

[2] Zhou S., Ye W., Zhang M., Liang J. (2012). The effects of nrf2 on tumor angiogenesis: A review of the possible mechanisms of action. Crit. Rev. Eukaryot. Gene Expr..

[3] Furfaro A.L., Traverso N., Domenicotti C., Piras S., Moretta L., Marinari U.M., Pronzato M.A., Nitti M. (2016). The Nrf2/HO-1 Axis in Cancer Cell Growth and Chemoresistance. Oxid. Med. Cell. Long..

[4] Ciamporcero E., Daga M., Pizzimenti S., Roetto A., Dianzani C., Compagnone A., Palmieri A., Ullio C., Cangemi L., Pili R. (2018). Crosstalk between Nrf2 and YAP contributes to maintaining the antioxidant potential and chemoresistance in bladder cancer. Free. Radic. Biol. Med..

[5] Daga M., Pizzimenti S., Dianzani C., Cucci M.A., Cavalli R., Grattarola M., Ferrara B., Scariot V., Trotta F., Barrera G. (2019). Ailanthone inhibits cell growth and migration of cisplatin resistant bladder cancer cells through down-regulation of Nrf2, YAP, and c-Myc expression. Phytomedicine.

[6] Cucci M.A., Grattarola M., Dianzani C., Damia G., Ricci F., Roetto A., Trotta F., Barrera G., Pizzimenti S. (2020). Ailanthone increases oxidative stress in CDDP-resistant ovarian and bladder cancer cells by inhibiting of Nrf2 and YAP expression through a post-translational mechanism. Free Radic. Biol. Med..

[7] Hayden A., Douglas J., Sommerlad M., Andrews L., Gould K., Hussain S., Thomas G.J., Packham G., Crabb S.J. (2014). The Nrf2 transcription factor contributes to resistance to cisplatin in bladder cancer. Urol. Oncol..

[8] Catanzaro E., Calcabrini C., Turrini E., Sestili P., Fimognari C. (2017). Nrf2: A potential therapeutic target for naturally occurring anticancer drugs?. Expert Opin. Ther. Targets.

[9] Zhu J., Wang H., Chen F., Fu J., Xu Y., Hou Y., Kou H.H., Zhai C., Nelson M.B., Zhang Q. (2016). An overview of chemical inhibitors of the Nrf2-ARE signaling pathway and their potential applications in cancer therapy. Free Radic. Biol. Med..

[10] Cai S.J., Liu Y., Han S., Yang C. (2019). Brusatol, an NRF2 inhibitor for future cancer therapeutic. Cell. Biosci..

[11] Harder B., Tian W., La Clair J.J., Tan A.-C., Ooi A., Chapman E., Zhang D.D. (2017). Brusatol overcomes chemoresistance through inhibition of protein translation. Mol. Carcinog..

[12] Ku S.H., Jo S.D., Lee Y.K., Kim K., Kim S.H. (2016). Chemical and structural modifications of RNAi therapeutics. Adv. Drug Deliv. Rev..

[13] Cavalli R., Primo L., Sessa R., Chiaverina G., di Blasio L., Alongi J., Manfredi A., Ranucci E., Ferruti P. (2017). The AGMA1 polyamidoamine mediates the efficient delivery of siRNA. J. Drug Target.

[14] Falzarano M.S., Argenziano M., Marsollier A.C., Mariot V., Rossi D., Selvatici R., Dumonceaux J., Cavalli R., Ferlini A. (2020). Chitosan shelled nanobubbles irreversibly encapsulate morpholino conjugate antisense oligonucleotides and are ineffective for PMO-mediated gene silencing. Nucleic Acid Ther..

[15] Cavalli R., Soster M., Argenziano M. (2016). Nanobubbles: A promising efficient tool for therapeutic delivery. Ther. Deliv..

[16] Leiro V., Santos S.D., Pego A.P. (2017). Delivering siRNA with Dendrimers: In Vivo Applications. Curr. Gene Ther..

[17] Weber N., Ortega P., Clemente M.I., Shcharbin D., Bryszewska M., de la Mata F.J., Gómez R., Muñoz-Fernández M.A. (2008). Characterization of arbosilane dendrimers as effective carriers of siRNA to HIV-infected lymphocytes. J. Control. Release.

[18] Sánchez-Nieves J., Fransen P., Pulido D., Lorente R., Muñoz-Fernández M.A., Albericio F., Royo M., Gómez R., de la Mata J.F. (2014). Amphiphilic cationic carbosilane-PEG dendrimers: Synthesis and application in gene therapy. Eur. J. Med. Chem..

[19] Ortega P., Sánchez-Nieves J., Martínez-Bonet M., Perisé-Barrios J., Gómez R., Muñoz-Martínez M.A., de la Mata F.J., Sangram K.S., Dubruelr P. (2015). Cationic Dendritic Systems as Non-viral Vehicles for Gene Delivery Applications. Cationic Polymers in Regenerative Medicine.

[20] Heredero-Bermejo I., Hernández-Ros J.M., Sánchez-García L., Maly M., Verdú-Expósito C., Soliveri J., de la Mata F.J., Copa-Patiño J.L., Pérez-Serrano J., Sánchez-Nieves J. (2018). Ammonium and guanidine carbosilane dendrimers and dendrons as microbicides. Eur. Polym. J..

[21] Clemente N., Argenziano M., Gigliotti C.L., Ferrara B., Boggio E., Chiocchetti A., Caldera F., Trotta F., Benetti E., Annaratone L. (2019). Paclitaxel-Loaded Nanosponges Inhibit Growth and Angiogenesis in Melanoma Cell Models. Front. Pharmacol..

[22] Schneider C.A., Rasband W.S., Eliceiri K.W. (2012). NIH Image to ImageJ: 25 years of image analysis. Nat. Met..

[23] Cucci M.A., Compagnone A., Daga M., Grattarola M., Ullio C., Roetto A., Palmieri A., Rosa A.C., Argenziano M., Cavalli R. (2019). Post-translational inhibition of YAP oncogene expression by 4-hydroxynonenal in bladder cancer cells. Free Radic. Biol. Med..

[24] Stetefeld J., McKenna S.A., Patel T.R. (2016). Dynamic light scattering: A practical guide and applications in biomedical sciences. Biophys. Rev..

[25] Ryan M.J., Johnson G., Kirk J., Fuerstenberg S.M., Zager R.A., Torok-Storb B. (1994). HK-2: An immortalized proximal tubule epithelial cell line from normal adult human kidney. Na+ dependent/phlorizin sensitive sugar transport; adenylate cyclase responsiveness to parathyroid, but not to antidiuretic, hormone. Kidney Int..

[26] Karleta V., Andrlik I., Braunmüller S., Franke T., Wirth M., Gabor F. (2010). Poloxamer 188 supplemented culture medium increases the vitality of Caco-2 cells after subcultivation and freeze/thaw cycles. ALTEX.

[27] Ortega P., Bermejo J.F., Chonco L., de Jesús E., de la Mata F.J., Fernández G., Flores J.C., Gómez R., Serramía M.J., Muñoz-Fernández M.A. (2006). Novel Water-Soluble Carbosilane Dendrimers: Synthesis and Biocompatibility. Eur. J. Inorg. Chem..

[28] Gonzalo T., Clemente M.I., Chonco L., Weber N.D., Díaz L., Serramía M.J., Gras R., Ortega P., de la Mata F.J., Gómez R. (2010). Gene Therapy in HIV-infected Cells to Decrease Viral Impact by Using an Alternative Delivery Method. ChemMedChem.

[29] Serramía M.J., Álvarez S., Fuentes-Paniagua E., Clemente M.I., Sánchez-Nieves J., Gómez R., de la Mata F.J., Muñoz-Fernández M.Á. (2015). In vivo delivery of siRNA to the brain by carbosilane dendrimer. J. Control. Release.

[30] Cavalli R., Bisazza A., Trotta M., Argenziano M., Civra A., Donalisio M., Lembo D. (2012). New chitosan nanobubbles for ultrasound-mediated gene delivery: Preparation and in vitro characterization. Int. J. Nanomed..

[31] Danaei M., Dehghankhold M., Ataei S., Hasanzadeh Davarani F., Javanmard R., Dokhani A., Khorasani S., Mozafari M.R. (2018). Impact of Particle Size and Polydispersity Index on the Clinical Applications of Lipidic Nanocarrier Systems. Pharmaceutics.

[32] Wakabayashi N., Chartoumpekis D.V., Kensler T.W. (2015). Crosstalk between Nrf2 and Notch signaling. Free Radic. Biol. Med..

[33] Tian Y., Liu Q., He X., Yuan X., Chen Y., Chu Q., Wu K. (2016). Emerging roles of Nrf2 signal in non-small cell lung cancer. J. Hematol. Oncol..

[34] Sova M., Saso L. (2018). Design and development of Nrf2 modulators for cancer chemoprevention and therapy: A review. Drug Des. Devel. Ther..

[35] Dzmitruk V., Apartsin E., Ihnatsyeu-Kachan A., Abashkin V., Shcharbin D., Bryszewska M. (2018). Dendrimers Show Promise for siRNA and microRNA Therapeutics. Pharmaceutics.

[36] Patravale V., Dandekar P., Jain R. (2012). Nanoparticulate Drug Delivery: Perspectives on the Transition from Laboratory to Market.

[37] Barba A.A., Bochicchio S., Dalmoro A., Lamberti G. (2019). Lipid Delivery Systems for Nucleic-Acid-Based-Drugs: From Production to Clinical Applications. Pharmaceutics.

[38] Santos A., Veiga F., Figueiras A. (2019). Dendrimers as Pharmaceutical Excipients: Synthesis, Properties, Toxicity and Biomedical Applications. Materials.

