# Supplementary material for: Carbosilane Dendrimers Loaded with siRNA Targeting Nrf2 as a Tool to Overcome Cisplatin Chemoresistance in Bladder Cancer Cells
Source: Antioxidants (Basel). 2020 Oct 14;9(10):993. doi: 10.3390/antiox9100993 (PMC7602517; doi:10.3390/antiox9100993)
Supplement: Supplementary file 1 [file antioxidants-09-00993-s001.pdf]

Ambrosio et al.
Carbosilane dendrimers loaded with siRNA targeting Nrf2 as a tool to overcome cisplatin chemoresistance in bladder cancer cells.

SUPPLEMENTARY MATERIALS

Fig 1S. CDDP-resistance and Nrf2 expression in bladder cancer cells

Both CDPP-resistance and Nrf2 expression was checked in T24 and 253J B-V C-r cells, to confirm our previously reported data (4). As shown in **Fig 1S**, panel A, 2.5 µg/ml CDDP did not affect cell viability (by MTT test) in both T24 and 253J B-V C-r at any time tested, while the inhibition in the sensitive 253J B-V parental clone was statistical significant since 24 h. Higher basal expression of Nrf2 protein in CDDP-resistant cells, with the respect to the sensitive 253J B-V parental clone, was also confirmed (**Fig. 1S, panel B**).

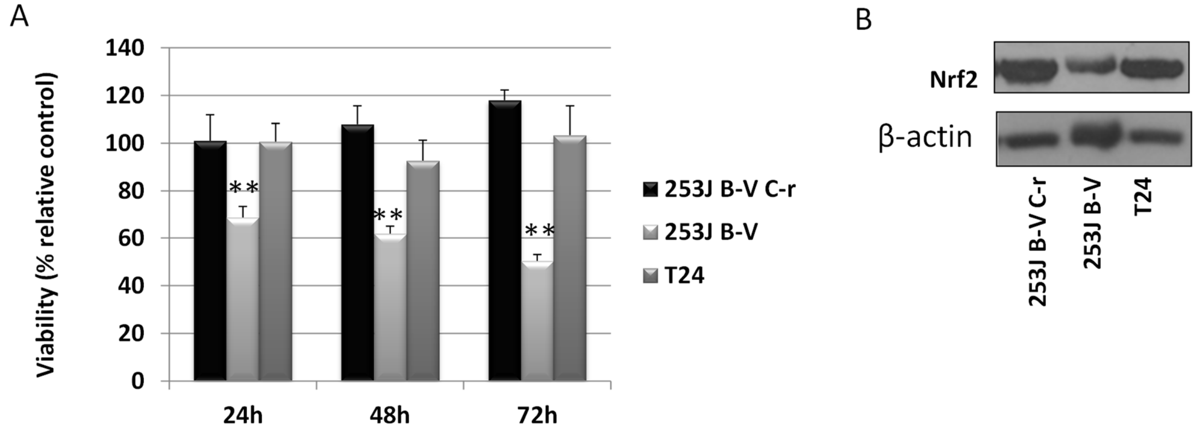

Fig. 1S Panel A: MTT assay in T24, 253J B-V C-r, 253J B-V cells treated with 2.5 µg/ml CDDP. Viability was checked at the indicated times. Results are expressed as percent of the relative control values and are the mean ± standard deviation of three separate experiments performed in triplicate. ** = p<0.01 versus relative control value. Panel B: Western blot analysis of Nrf2 in T24, 253J B-V C-r, and 253 J B-V. Equal protein loading was confirmed by exposure of the membranes to the anti-GAPDH antibody.

Fig. 2S. Nrf2 ~~and GstA4~~ inhibition with a lower amount of siNrf2-GCD

T24 and 253J B-V C-r cells were treated with siNrf2-GCD alone (final concentrations 4 μg/ml GCD, 0.04 μM siNrf2) or with naked siNrf2 4 μg/ml transfected the HiPerFect® reagent. Results showed that a lower amount of siNrf2-GCD was able to inhibit Nrf2.Transfection with the HiPerFectR reagent, used as a positive control, showed similar inhibitions.

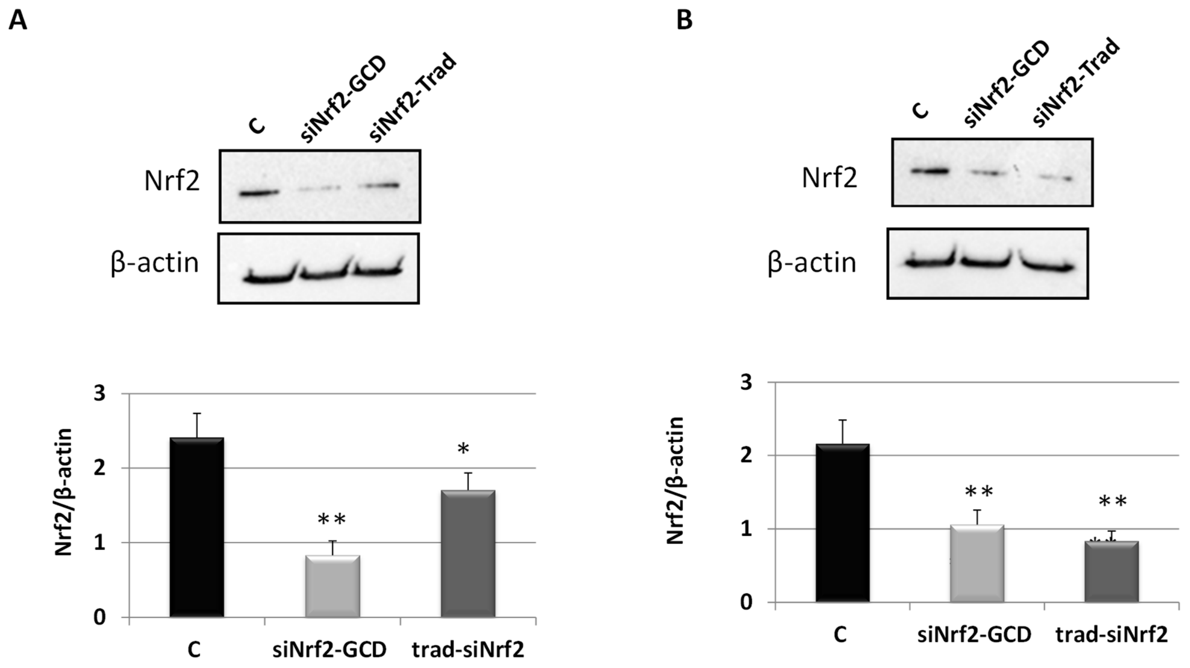

Fig. 2S Western blot analysis of Nrf2 in T24 (A) and 253 J B-V C-r CDDP-resistant cells (B), untreated (control, C), treated with siNrf2-GCD (0.04 μM siNrf2 in 4 μg/ml GCD), transfected with the same amount of siNrf2 (0.04 μM) in HiPerFect® reagent with the traditional method (siNrf2-Trad). Cells were collected after 24 h from the treatments. Below, relative densitometric scanning of Nrf2 expression normalized using the β-actin signal. Data are the mean ± SD from three independent experiments. **p≤0.01, *p≤0.05 vs. control.
